# The impact of alpha-1 antitrypsin augmentation therapy on neutrophil-driven respiratory disease in deficient individuals

**DOI:** 10.2147/JIR.S156405

**Published:** 2018-03-26

**Authors:** Danielle M Dunlea, Laura T Fee, Thomas McEnery, Noel G McElvaney, Emer P Reeves

**Affiliations:** Irish Centre for Genetic Lung Disease, Department of Medicine, Royal College of Surgeons in Ireland, Beaumont Hospital, Dublin, Ireland

**Keywords:** neutrophils, alpha-1 antitrypsin deficiency, alpha-1 antitrypsin augmentation, inflammation, airways disease

## Abstract

Alpha-1 antitrypsin (AAT) is the most abundant serine protease inhibitor circulating in the blood. AAT deficiency (AATD) is an autosomal codominant condition affecting an estimated 3.4 million individuals worldwide. The clinical disease associated with AATD can present in a number of ways including COPD, liver disease, panniculitis and antineutrophil cytoplasmic antibody vasculitis. AATD is the only proven genetic risk factor for the development of COPD, and deficient individuals who smoke are disposed to more aggressive disease. Principally, AAT is a serine protease inhibitor; however, over the past number of years, the assessment of AAT as simply an antiprotease has evolved, and it is now recognized that AAT has significant anti-inflammatory properties affecting a wide range of cells, including the circulating neutrophil.

## Introduction

Alpha-1 antitrypsin (AAT) is a member of the serpin family which also includes plasminogen activator inhibitor-1, alpha-1 antichymotrypsin, antithrombin and C1-inhibitor. These serpins play vital roles in the regulation of proteases involved in fibrinolytic, complement and coagulation pathways.^1^ AAT is a 394-amino acid polypeptide chain encoded by the SERPINA1 gene located at the chromosomal region 14q32.1.^2^ Aside from hepatocytes where it is mostly synthesized, AAT is also produced to a lesser degree by other cell types such as neutrophils,^4^ macrophages,^3^ monocytes,^5^ intestinal epithelial cells,^6^ pancreatic islets^7^ and cancer cells.^8^ However, from these cellular sources, the AAT protein is unlikely to contribute to circulating plasma levels but rather to local AAT concentrations.^9^ Within the circulation, the concentration of AAT is 1.21–2.17 g/L, making it one of the most abundant plasma proteins with a half-life of 4.6 days.^10^ AAT is part of the acute-phase response, which means that a rapid rise in plasma levels of AAT is observed during acute inflammation,^11^ with plasma levels increasing three- to four fold.^12^ The aim of this review is to first introduce AAT deficiency (AATD) and then to consider the described anti-inflammatory activities of AAT in controlling key neutrophil functions, outline recognized signaling pathways and specifically recognize the features of neutrophil-driven airways disease in which AAT augmentation therapy has been demonstrated to be effective. Review of the literature was carried out using the MEDLINE (from 1986 to 2017), Google Scholar and The Cochrane Library databases.

## The antiprotease AAT

**Figure 1:**
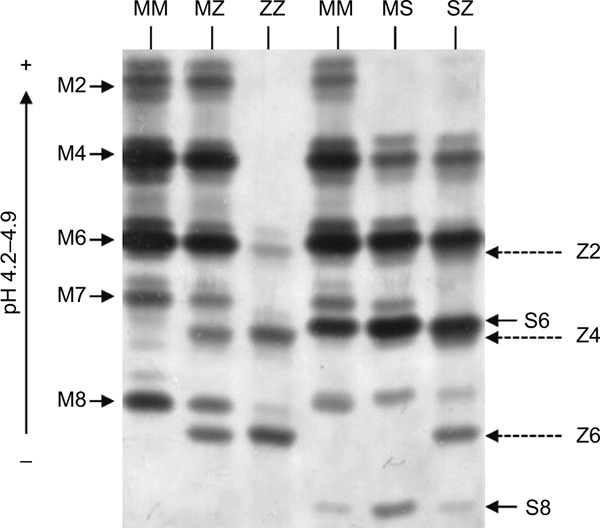
Isoelectric focusing gel illustrating AAT phenotype mutations. The glycan numbers for the phenotypes are labeled. **Abbreviation:** AAT, alpha-1 antitrypsin.

The predominate role of AAT is as a serine protease inhibitor, chiefly inhibiting neutrophil elastase (NE),^13^ but also other proteases including chymotrypsin, cathepsin G (CathG), proteinase 3 (PR3) and thrombin. The structure of the AAT is critical for its antiprotease activity and comprises 3 beta sheets (A, B and C), 9 alpha helices and a reactive center loop (RCL) at the C-terminal end.^14^ Furthermore, during AAT production, posttranslational modifications occur, and the protein undergoes addition of *N*-linked oligosaccharides at asparagines 70, 107 and 271. The three *N*-glycosylation sites on the AAT molecule contain mostly biantennary structures but also triantennery and traces of tetraantennary *N*-glycans.^15^ Multiple glycoforms of AAT have been identified (M0–M8), and these can be visualized by isoelectric focusing (IEF) and separated by the charge of the *N*-glycans (Figure 1). Adding to this field, we have recently published that during the acute inflammatory process of community-acquired pneumonia (CAP), the circulating AAT molecule differs due to variations in its glycosylation pattern and that AAT glycans containing 4 sialic acids appeared during the resolution phase of CAP.^16^ Moreover, data highlight the role of sialylation in the anti-inflammatory activity of AAT, as during the resolving phase of infection there was a significant increase in circulating levels of interleukin (IL)-8 complexed to sialylated negative glycoforms of AAT. This binding event led to enhanced inhibition of C-X-C motif chemokine receptor (CXCR) 1 engagement on neutrophil plasma membranes,^16^ which may serve to prevent further migration of cells to epithelial surfaces and decrease the potential for neutrophil-mediated damage.

The antiprotease inhibitor activity of the molecule lies within the 9-amino acid RCL. AAT, unlike most proteins, folds into a metastable state which has a considerably lower conformational stability.^17^ Fundamentally, the AAT molecule acts as a trap with the RCL as its bait. NE cleavage between amino acids 358 and 359 of the RCL results in the creation of an AAT:NE complex between the cleaved AAT molecule and NE. The process results in irreversible inactivation of both molecules, and thus, in the ideal scenario, AAT exists in the lungs surplus to the amount of protease in order to protect the lung parenchyma from degradation. Moreover, the structural rearrangement that enables the AAT:NE complex to form exposes a binding site that can engage with a receptor known as SERPIN:enzyme complex receptor. The interaction of this AAT:NE complex with the SERPIN:enzyme complex receptor on cell surfaces such as hepatocytes causes a positive feedback loop leading to increased expression of the SERPINA1 gene.^18^

## AAT deficiency

AATD is an autosomal, codominant, genetic disorder that is characterized by low circulating levels of AAT as a result of a mutation of the SERPINA1 gene. The worldwide frequency of AATD varies according to population and is particularly prevalent in Europe with multiple studies reporting a high prevalence of the deficiency alleles in Poland,^19^,^20^ France,^21^ Italy^22^ and Ireland.^23^ AATD is characterized by circulating levels <11 µmol/L, which is the putative protective threshold level.^24^ The AAT phenotype is determined by codominant expression of parental alleles with the majority of individuals carrying 2 copies of non-mutated M allele. The M allele in homozygous individuals leads to AAT plasma levels >1.04 g/L or 20 µM. However, the SERPINA1 gene is highly pleomorphic, and at least 120 genetic variants have been reported to date,^25^ with the Z (Glu342Lys) and S (Glu264Val) mutations (Figure 1) being the most common. The substitution of glutamic acid for lysine at position 342 leads to the Z mutation, and that of glutamic acid for valine at position 264 gives rise to the S mutation.^26^,^27^

In individuals heterozygous for the S mutation, AAT levels typically remain above the protective threshold of 11 µM. This mutation is thus considered to carry a negligible risk of AATD-associated disease unless co-inherited with another deficiency allele such as the Z allele. The Z mutation gives rise to the most severe plasma deficiency and occurs in more than 95% of individuals with AATD.^28^ Z-AAT protein polymerizes and becomes trapped within the endoplasmic reticulum (ER), thus accumulating in hepatocytes. This results in impaired secretion of the protein,^29^ leading to plasma deficient in AAT, with individuals homozygous for the Z mutation having 10%–15% of normal circulating levels. It is because of these low circulating levels of AAT that patients with this condition are at a high risk of developing emphysema.^30^ The low level of AAT in ZZ individuals results in an imbalance of proteases and antiproteases in the AATD lung, resulting in unchecked levels of active serine proteases that damage alveolar tissue leading to lung disease.^28^,^31^

Another class of mutations in the SERPINA1 gene are termed silent or “null” mutations. The plasma levels of this class of variants are undetectable by conventional techniques such as nephelometry and IEF, and as such, these mutations are classically thought to result in a complete absence of AAT production and therefore a high risk of developing emphysema.^32^ While the consequence of the null alleles is unified, that is, undetectable plasma levels of AAT, the mutational events from which they arise vary from gene deletions, premature stop codon insertions, to mRNA degradation. For example, the Q0_granite_ falls genotype arises from a single base pair deletion, resulting in a premature stop codon and a lack of mRNA production.^33^

**Figure 2:**
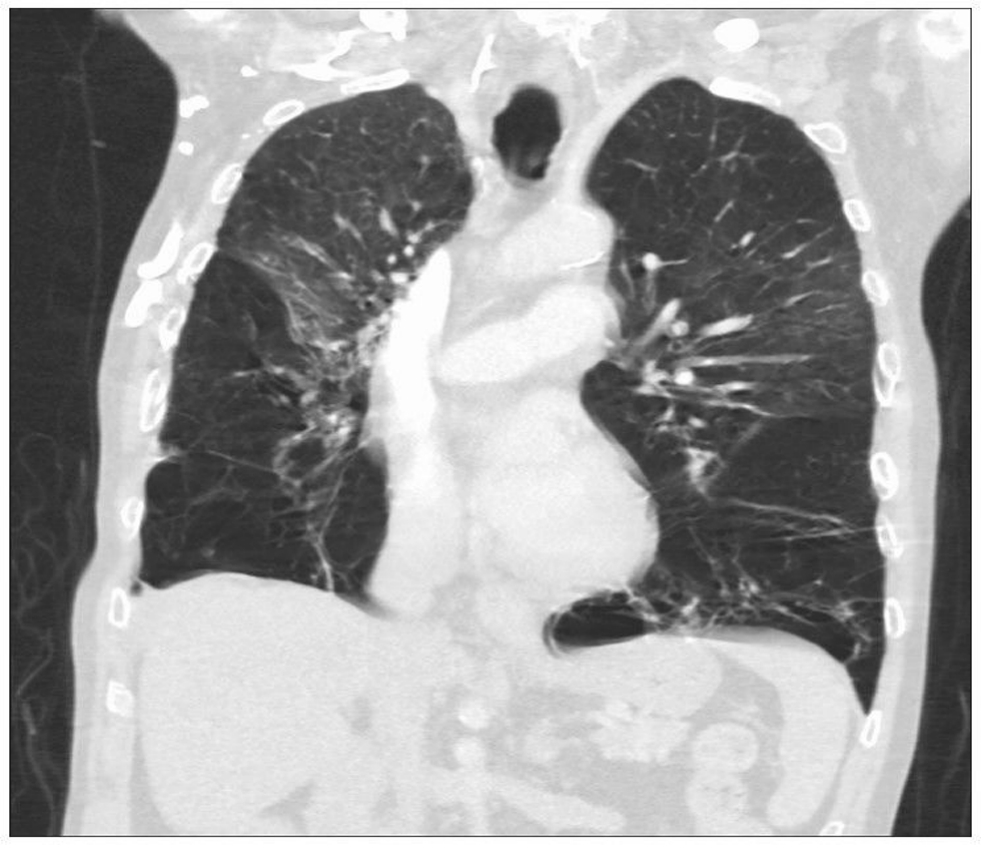
A computed tomography scan showing severe bilateral, lower lobe-predominant panacinar emphysema in a patient with AATD homozygous for the Z mutation. **Abbreviation:** AATD, alpha-1 antitrypsin deficiency.

The classical manifestation of AATD in the lungs is panacinar^34^ and lower lobe-predominant emphysema, involving the distal airway structures and resulting in uniform enlargement of the bronchioles and alveoli (Figure 2). The clinical presentation of COPD in AATD is similar regardless of AAT phenotype, with patients commonly complaining of dyspnea and cough with frequent respiratory infections. Onset occurs at a significantly younger age in AATD patients however, often in the 3rd to the 4th decade of life. This is in contrast to typical COPD, which usually involves upper lobe-predominant centrilobular emphysema^35^ that commonly manifests in the late 6th or 7th decade.^30^

The risk for emphysema is increased in individuals heterozygous for the Z mutation who are smokers.^29^ Smoking has been demonstrated to further exacerbate the imbalance between proteases and antiproteases by rendering AAT inactive.^36^,^37^ Increased release of NE in bronchoalveolar lavage fluid (BALF)^38^ in patients who smoke has been reported, and a direct correlation has been made between the NE burden in the BALF and the degree of emphysema seen on computed tomography scans.^40^ Moreover, the inverse relationship between the degree of emphysema and the antielastase activity in BALF of COPD patients further supports the protease/antiprotease theory of emphysema.^41^ In addition to the increased release of proteases, another process that can occur which further contributes to airway inflammation is oxidative stress. The conversion of hydrogen peroxidase to its product hypochlorous acid by myeloperoxidase, along with other reactive oxygen species, can render AAT inactive through oxidation and chlorination.^42^ Hydrogen peroxidase, a component of cigarette smoke, oxidizes 2 methionine residues, 351 and 358, located on the RCL of AAT. The outcome of AAT oxidation is loss of anti-NE capacity.

The traditional view behind the cause of AATD-related emphysema is that it is a result of the imbalance of proteases and antiproteases in the lung. However, it has recently come to light that AAT is more than just an antiprotease. AAT has been shown to have anti-inflammatory capacities outside of its antiprotease activity. The loss of this AAT function is apparent as the manifestations of AATD are not confined to lung disease but extend to systemic inflammatory conditions such as vasculitis^43^ and panniculitis,^44^ which will be discussed below. Therefore, disease manifestations seen in AATD are due to loss of AAT function as both a protease inhibitor and an anti-inflammatory molecule.

## The neutrophils in the pathogenesis of AATD disease

Neutrophils are the source of NE, CathG and PR3, the key drivers of inflammation that destroy alveolar tissue in AATD. Published data have demonstrated an increased number of these polymorphonuclear leukocytes in the BALF of AATD patients.^45^ These cells are produced from myeloid precursors in the bone marrow and have a short circulating half-life of ~8 hours.^46^ Neutrophils are typically the first leukocytes that migrate to the site of inflammation, exiting the bloodstream where they transmigrate through the endothelium and travel to the site of infection. Neutrophil localization to the infected site is crucial for the clearance of infection, as a defective neutrophil response has been shown to lead to bacterial colonization.^47^

Upon arrival at the site, the activated neutrophil has an increased life span to ensure microbial clearance.^48^ The neutrophil’s arsenal of antimicrobial serine proteases is stored within primary granules. Upon neutrophil activation, there is a rapid translocation of primary granules to the plasma or phagocytic membrane, thereby releasing serine proteases including NE, CathG and PR3 into the extracellular or phagocytic space where they play an important role in host defence.^49^,^50^ For example, NE is essential in the killing of Gram-negative bacteria, as evidenced by the finding that NE-knockout mice are more susceptible to these microbes.^51^ Conversely, besides playing a protective role against invading microorganisms, these proteases have been associated in the pathogenesis of emphysema and COPD.^52^,^53^ Indeed, NE is referred to as a double-edged sword as unchecked levels can degrade a wide variety of host substrates. In AATD, NE is considered to be the major protease involved in the destruction of lung tissue as it possesses the ability to damage every component of the extracellular matrix including cross-linked fibrin, collagen and proteoglycans.^54^

Additionally, NE amplifies the inflammatory burden by stimulating mucin secretion yet decreasing the beat frequency of cilia of bronchial epithelial cells, thereby interrupting mucociliary clearance.^55^,^56^ Studies in mice have shown that NE augments the activity of further destructive proteases, including matrix metalloprotease (MMP) 9.^52^ Furthermore, NE also retains the capability to inactivate the innate inhibitors of these proteases including tissue inhibitors of metalloproteinases 1 and 2, inhibitors of MMP-9^57^ and MMP-2^58^ and other relevant protease inhibitors such as elafin^59^ and secretory leukocyte protease inhibitor.^60^

**Figure 3:**
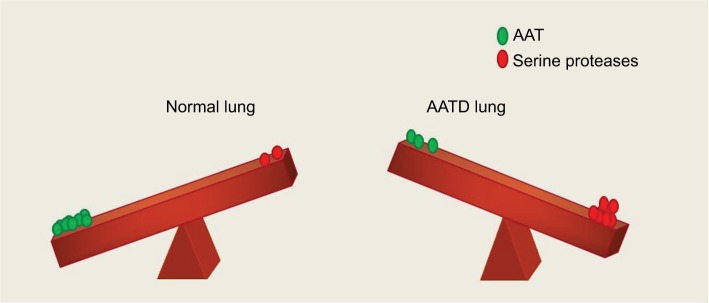
Protease/antiprotease balance shown in the lung of a healthy individual and a person with AATD. In a normal individual, the lung parenchyma is protected from serine protease activity of NE, CathG and PR3 by AAT. In an AATD individual, unchecked levels of proteases damage lung tissue due to low levels of AAT. **Abbreviations:** AAT, alpha-1 antitrypsin; AATD, AAT deficiency; CathG, cathepsin G; NE, neutrophil elastase; PR3, proteinase 3.

By proteolysis of complement receptors^61^,^62^ and CXCR1^63^ on neutrophils, NE quickly impairs the capacity of neutrophils to kill invading microbes. NE also weakens innate immunity by cleaving TIM-3 (T-cell Ig and mucin domain-containing molecule-3) from epithelial and neutrophil cell surfaces,^64^,^65^ and humoral immunity by cleaving immunoglobulins.^66^ NE destroys signaling cytokines including the interferon-gamma (IFN-γ)-inducing factor IL-18,^67^ thus potentiating the airway inflammatory environment. Adding to the proteolyic burden, CathG digests host substrates and is seen at higher levels in patients with emphysema.^52^ There is thus a tremendous need to curtail the excessive activity of both NE and CathG, and by binding to them, AAT inhibits the destructive nature of these proteases. Indeed, AAT is a regulator of NE,^68^ CathG^69^ and PR3,^70^ and it is for this reason that the balance created by AAT is essential for protection of the lung matrix (Figure 3). During pulmonary exacerbations, the sputum of AATD patients has been found to have increased levels of PR3. As the elastolytic ability of PR3 is less than NE, PR3 likely plays a lesser role in the manifestation of emphysema.^71^ In contrast, however, anti-PR3 autoantibodies have been proposed to exacerbate the degranulation process by binding PR3 expressed on surface membranes of monocytes and neutrophils, resulting in excess protease release and contributing to both vascular and endothelial injuries.^71^,^72^

**Figure 4:**
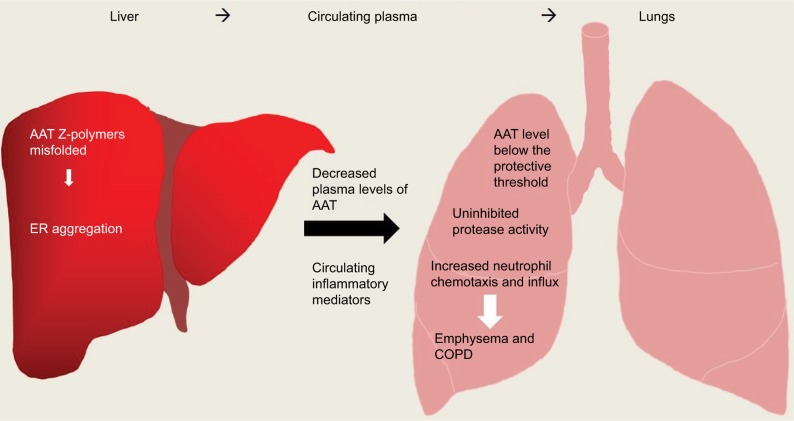
Overview of inflammation caused in AATD liver and lung disease. AAT Z-polymers misfold in the liver leading to retention/aggregation in the ER, resulting in low levels of plasma AAT. Low levels of AAT and active uninhibited serine proteases can cause damage to lung parenchyma ultimately leading to emphysema and COPD. **Abbreviations:** AAT, alpha-1 antitrypsin; AATD, AAT deficiency; ER, endoplasmic reticulum.

Studies aimed at understanding the increased neutrophil burden seen in AATD patients have demonstrated that individuals either homozygous or heterozygous for the Z allele have an increased influx of neutrophils into their lungs.^73^ A number of models have been proposed to help explain this increase, and one suggestion is the deposition of polymers of mutated Z-AAT protein. Polymerization of the Z-AAT protein occurs mainly within hepatocytes but is also thought to occur spontaneously in the lungs of AATD individuals. In ZZ-AATD individuals, these polymers have been detected in BALF^74^ and additionally in the alveolar walls of individuals with emphysema.^75^ The presence of polymerized Z-AAT induces a pro-inflammatory response as these polymers can act as a chemoattractant, inducing chemotaxis levels comparable to IL-8^76^ and complement component C5a (Figure 4).^77^ Z-AAT polymers have also been reported to stimulate neutrophil adhesion and induce neutrophil degranulation.^77^ Extending on this concept, while many investigators agree that mutations of the AAT protein can lead to the formation of Z-AAT polymers that are retained within the ER of hepatocytes in ZZ-AATD,^78^,^79^ the characteristics of Z-AAT polymers and ER stress in immune cells from ZZ-AATD patients are less well studied. In neutrophils, an intrinsic defect due to misfolded AAT protein within the ER of circulating ZZ-AATD cells results in increased expression of the proapoptotic transcription factor CHOP, with accelerated apoptosis of ZZ-AATD neutrophils associated with decreased bacterial killing.^80^

When describing neutrophil-dominated inflammation with regard to AATD, it is important to discuss panniculitis. This rare condition (occurring in 0.1% of Z homozygotes)^81^ is characterized by intense neutrophil infiltrates in the skin, presenting as a painful skin rash.^81^ It has been described most commonly in ZZ patients but has also been found in MZ and SZ individuals.^81^ The pathophysiology of AATD-related panniculitis has not been definitively described, but skin biopsies demonstrate neutrophil infiltration into the subcutaneous tissues and resultant tissue destruction due to the low levels of antiprotease and high levels of protease. The presence of polymers of Z-AAT in the skin is significant,^82^ as these have been shown to be a powerful neutrophil attractant, as mentioned earlier.^75^

## The effect of AAT on neutrophil function

IL-8 is a commanding neutrophil chemoattractant produced by airway epithelial cells and alveolar macrophages in response to inflammation^83^ and has been shown to be increased in the sputum of MZ heterozygous individuals.^84^ IL-8 engages with CXCR1 on the neutrophil membrane, resulting in amplified neutrophil adhesion due to increased membrane expression of CD11b and CD18.^85^ The engagement of IL-8:CXCR1 also causes a rise in intracellular calcium levels, which facilitates neutrophil cytoskeletal rearrangements ultimately enabling chemotaxis.^86^ It has been shown that NE can induce the expression of this chemokine in bronchial epithelial cells via Toll-like receptor 4, thus adding to the inflammatory burden in the lung.^87^ In a similar fashion, unopposed protease activity results in the activation of protease-activated receptors (PARs). PARs 1, 2, 3 and 4 have all been found in the lung,^88^,^89^ and in the absence of AAT, these PARs are overactivated by neutrophil serine proteases, thus amplifying inflammation.

In recent years, it has emerged that AAT possesses a variety of anti-inflammatory properties. In this regard, it has been found that AAT can modulate IL-8-induced chemotaxis by binding this chemokine. At physiological pH, AAT protein has an overall negative charge that enables electrostatic interaction between AAT and positively charged IL-8. The oligosaccharides on AAT are vital for this anti-inflammatory function as it has been shown that non-glycosylated AAT fails to bind IL-8.^90^ This binding event between AAT and IL-8 impedes docking of IL-8 with CXCR1,^90^ impacting negatively upon the downstream signaling events involved in cytoskeleton rearrangement, F-actin formation and calcium flux, ultimately resulting in decreased neutrophil migration.

AAT has also been shown to influence neutrophil chemotaxis in response to soluble immune complexes (sICs). Neutrophil engagement of sICs results in increased tumor necrosis factor-alpha (TNF-α)-converting enzyme (TACE) activity, causing release of the glycosylphosphatidylinositol-anchored Fc receptor (FcγRIIIB), which is a prerequisite for chemotaxis. AAT was shown to modulate TACE activity, thereby preventing the release of membrane FcγRIIIB.^90^ Moreover, the ability of AAT to reduce neutrophil chemotaxis in response to a third stimuli, namely, formyl-methionyl-leucyl-phenylalanine, with subsequent reduced adherence to lung-derived endothelial cells has been demonstrated.^91^ Furthermore, leukotriene B_4_ (LTB_4_) is another stimulant that can affect neutrophil function, increasing cell adhesion, degranulation and chemotaxis.^92^–^94^ In a vicious circle of inflammation, released NE can signal back to the neutrophil causing increased production of LTB_4_ and upregulation of its receptor BLT1 on the neutrophil membrane. In turn, in vitro AAT has been reported to exercise strong anti-inflammatory effects against LTB_4_, binding this lipid mediator via a central hydrophobic pocket on the protein surface. This binding event inhibits the engagement of LTB_4_ with BLT1 on the neutrophil plasma membrane, thereby preventing neutrophil activation (Figure 4).^94^

With regard to degranulation, in the neutrophil-burdened airways, there is an exuberant release of cytotoxic granule proteins to the outside of the cell. In landmark studies, degranulation of all granule subtypes in response to either TNF-α or LTB_4_ was significantly reduced by AAT. In this regard, Bergin et al reported a substantial rise in TNF-α membrane expression in circulating neutrophils isolated from AATD individuals compared to healthy controls, on par with levels in patients with rheumatoid arthritis.^95^ The authors suggested that AAT may in turn control TNF-α bioactivity and therefore could reduce neutrophil degranulation in response to TNF-α.^96^ Ensuing in vitro results demonstrated that AAT modulates degranulation of neutrophil secondary and tertiary granules and that the inhibitory mechanism involves the ability of AAT to bind TNF receptors (TNFRs), thereby blocking TNF-α engagement with TNFR1 and TNFR2.^95^ This blockade of receptor engagement led to the prevention of downstream signaling events including MAPK p38 phosphorylation, a key step in the neutrophil degranulation process.^97^ This is further supported by in vivo and in vitro data on the ability of AAT to modulate TNF-α-induced apoptosis.^98^,^99^ AAT’s capacity to inhibit ligand–receptor binding with resultant reduced downstream signaling events has also been described for transferrin.^100^ Collectively, these studies strongly support the vital role that AAT plays in reducing the neutrophil burden in the airways and protecting lung tissue architecture from destruction, as seen in AATD.

## The implications of augmentation therapy for neutrophil function in AATD patients

It is evident that the protease/antiprotease balance in the lung is of great importance in maintaining healthy tissue. Therefore, a logical treatment of AATD-related lung disease is to reestablish physiological concentrations of AAT. The current gold standard treatment for AATD is augmentation therapy utilizing AAT sourced from pooled human plasma at a dose of 60 mg/kg, administered by slow intravenous infusion once weekly. In the early 1980s, it was shown that intravenous infusion of purified human AAT corrected the concentration of AAT in plasma and on the lung epithelial surface which was used as a surrogate for lung parenchyma.^101^,^102^ It has been subsequently proven that AAT augmentation therapy ameliorates AATD-related lung disease, with the RAPID (Randomized, Placebo-controlled Trial of Augmentation Therapy in Alpha-1 Proteinase Inhibitor Deficiency) trial being the first to conclusively demonstrate the benefit of AAT augmentation therapy as compared to placebo.^103^ The extension to this trial, RAPID-OLE (RAPID open-label extension), further supported the continued efficacy of AAT in decelerating the progression of AATD lung disease over 4 years.^104^

Perhaps surprisingly, the RAPID trial did not demonstrate a significant reduction in frequency of exacerbations with AAT augmentation therapy.^103^ Exacerbations are commonly neutrophil-driven events characterized by worsening of dyspnea and cough with increased sputum production. There is some evidence for a reduction in exacerbation frequency with augmentation therapy,^105^ but this is a notoriously difficult outcome to capture, and thus, inadequate power could be an explanation for the lack of benefit observed. Further anecdotal evidence for a benefit comes from surveys amongst AATD patients, reporting a decrease in both chest infections and hospitalizations in association with augmentation therapy.^106^

These results lend weight to the clinical use of AAT augmentation therapy for airways disease, which is currently available in the US, Canada and several European countries including Spain, Italy and Germany. Intravenous augmentation with AAT is also used as a treatment for panniculitis since the first report in 1987 in 2 ZZ patients whose skin condition had proven refractory to conventional therapy.^107^ Both demonstrated remarkable improvement with augmentation therapy. Subsequently, numerous case reports support the use of this therapy, commonly at a higher dose of 120 mg/kg weekly.^81^ Occasionally, panniculitis may coincide with a marked neutrophilic serositis manifesting as pleural effusions or arthritis and also characterized by dramatic improvement following AAT augmentation therapy.^108^ Successful treatment of nonspecific vasculitis in an AATD patient with intravenous augmentation therapy has also been described.^109^

A number of studies have specifically investigated the effect of AAT augmentation therapy on neutrophil dysfunction seen in AATD. Bergin et al found that in clinically stable ZZ patients, the neutrophils have increased levels of TACE activity on their membranes, leading to a higher chemotactic index.^90^ Post AAT augmentation therapy, the increase in plasma concentration of AAT resulted in normalized ZZ-AATD neutrophil chemotactic responses by inhibiting TACE activity and thereby preventing FcγRIIIB from being shed from the cell membrane, reducing it to that of healthy control levels. Further work demonstrated that augmentation therapy restored AAT plasma levels and normalized TNF-α signaling, thereby preventing TNF-α-induced neutrophil release of secondary and tertiary granules and resultant production of autoantibodies.^80^ Additionally, it was recently reported that AATD is associated with increased neutrophil membrane-bound NE, which can trigger an inflammatory cycle inducing secretion of LTB_4_ that further stimulates primary granule release. Overall, these findings highlight a novel interplay between LTB_4_ and NE released from neutrophils. In vivo plasma levels of both LTB_4_ and neutrophil membrane-bound NE were reduced in AATD patients receiving AAT augmentation therapy, compared with untreated patients matched by forced expiratory volume in 1 second and in fact normalized to healthy control MM levels. Relevant to the latter findings, a reduction in airway levels of IL-8 and LTB_4_ in individuals with cystic fibrosis (CF) treated with aerosolized AAT has been reported,^93^ suggesting the use of AAT outside of the context of AATD.

Moving to the end of the neutrophil’s life cycle, AAT augmentation therapy has been reported to impact upon neutrophil apoptosis. In AATD patients receiving AAT augmentation therapy, reduced TACE activity and TNF-α signaling and normalized neutrophil apoptosis were reported.^80^ AAT also binds to and inhibits caspase-3, thereby preventing lung endothelial cell apoptosis.^111^ Additionally, although not specific to neutrophils, AAT prolonged allograft survival and modulated cellular immunity in treatment of mice that had undergone pancreatic islet allograft.^99^,^112^–^114^ An investigation into how AAT protects islets cells revealed that AAT potentiated insulin secretion and the effects of glucagon-like peptide-1 and forskolin.^115^ Furthermore, AAT was shown to protect a diabetic cell line from TNF-α-mediated apoptosis and to significantly reduce apoptosis caused by a combination of TNF-α, IL-1β and IFN-γ.^115^

## Positive effects of AAT as treatment in other lung diseases

The increasing recognition of the immune-modulatory effects of AAT, alongside its broad antiprotease activity, has led to its consideration as a therapeutic agent in a wide range of conditions characterized by injurious neutrophilic inflammation,^116^ most prominently in CF. CF is the most commonly inherited fatal disease in Caucasians. It results from mutations in the CFTR gene that codes for an ion channel present on the lung epithelial membrane (amongst other tissues). Mutation results in viscous respiratory mucus that cannot be cleared from the lung. Chronic respiratory infection ensues, ultimately resulting in bronchiectasis or permanent enlargement of the proximal airways with destruction of their walls. Neutrophils are centrally implicated in this condition, with increased levels of NE suggesting a relative deficiency of AAT in the lung,^117^ leading to the evaluation of AAT as a potential therapy in CF. A clinical trial of aerosolized AAT at a dose of 1.5–3 mg/kg twice daily for 1 week demonstrated safety and tolerability as well as inhibition of airway NE and improved clearance of bacteria.^118^ A subsequent study extended therapy to 4 weeks, demonstrating a reduction in airway neutrophils.^110^ In contrast, however, a Phase II trial examining the effect of non-glycosylated recombinant AAT demonstrated safety and tolerability but showed a limited effect on NE activity and other markers of inflammation.^119^ A similar result showed decreased taurine, a surrogate for neutrophils, with no change in NE activity.^120^ These discrepancies may be caused by a number of reasons including but not limited to the glycosylation state of the AAT protein, different aerosol devices and methods of sampling CF airways. This remains an active area of research with randomized placebo-controlled trials in progress.

Another condition in which AAT is being investigated as a therapy in humans is bronchiolitis obliterans syndrome (BOS), a subtype of chronic rejection following lung transplant that is characterized by neutrophilic and lymphocytic inflammation and fibrosis of small airways.^121^ There is a theoretical basis and some supporting data for the potential benefit of AAT in other conditions characterized by excessive neutrophilic inflammation such as inflammatory bowel disease, rheumatoid arthritis and postoperative systemic inflammatory response syndrome (SIRS).^122^ For example, Daemen et al have demonstrated that AAT mitigates renal reperfusion injury in mice via reduced TNF-α and neutrophil influx.^123^ Kaner et al have shown that transgenic mice expressing the human AAT gene have reduced levels of liver and pancreatic dysfunction compared to wild-type mice in an SIRS model, as well as improved survival at 24 hours.^124^ Studies on human pancreatic beta cells in vitro and in mice have suggested a protective and regenerative effect of AAT on these insulin-producing cells, with potential implications in the treatment of autoimmune diabetes mellitus.^99^,^125^ Likewise, AAT has been shown to prolong survival of transplanted beta cells in mice^112^ and monkeys^126^ and ameliorate graft-vs-host disease in a mouse model.^127^ These results are the basis for several ongoing clinical trials in humans. In summary, AAT also has numerous effects on a range of cell types including monocytes, B cells, T cells, dendritic cells and macrophages, both directly and indirectly. These effects have been reviewed in detail elsewhere^116^,^128^–^129^ and further strengthen the concept of AAT as an important modifier of immune and inflammatory responses. Further understanding of how AAT interacts with the neutrophil, as well as other immune cells, may expand its use as a therapeutic agent outside of the setting of genetic AATD.

## Conclusion

The lungs of an AATD individual are burdened with high levels of proteolytic agents including NE, CathG and PR3 as a result of neutrophilic inflammation (Figure 3). The primary function of AAT as a protease inhibitor protecting the lung parenchyma from these destructive proteases is apparent in AATD as the diminished AAT levels in these individuals enable these proteases to go unchecked. However, recent work has shown that this multifaceted protein exerts more than just an antiprotease function in the circulation and lung. It has been found that AAT possesses anti-inflammatory properties independent of its antiprotease activity, bringing us away from the classical view of AAT. These anti-inflammatory properties are fundamental when trying to understand the manifestation of both the lung and systemic inflammation that is seen in AATD. The AATD neutrophils have dysregulated neutrophil adhesion, chemotaxis, degranulation and apoptosis. The ability of AAT to correct this dysregulation is seen following augmentation therapy, as AAT binds a number of pro-inflammatory mediators via hydrophobic and electrostatic interactions resulting in normalized neutrophil responses. This demonstrates that AAT exerts a wide array of immune-modulating effects, illustrating the wider role it plays, rather than just the protease inhibitor it was once thought. Nevertheless, a number of challenges remain in the development of AAT as an anti-inflammatory therapy such as expanding our knowledge of its mode of action and the development of new sources of glycosylated AAT with equivalent anti-inflammatory capacity to that of plasma purified protein. Despite these challenges, AAT holds incredible potential as a novel anti-inflammatory molecule, which has already been established as a safe and well-tolerated therapeutic agent.
